# mSphere of Influence: Translating Gut Microbiome Studies To Benefit Human Health

**DOI:** 10.1128/mSphere.00592-20

**Published:** 2020-07-08

**Authors:** Anna M. Seekatz

**Affiliations:** a Department of Biological Sciences, Clemson University, Clemson, South Carolina, USA

**Keywords:** infectious diseases, microbiome, microbiota

## Abstract

Anna M. Seekatz works in the field of the gut microbiome as it related to infectious diseases. In this “mSphere of Influence” article, she reflects on how two studies, “The impact of a consortium of fermented milk strains on the gut microbiome of gnotobiotic mice and monozygotic twins” (N. P. McNulty, T. Yatsunenko, A. Hsiao, et al., Sci Transl Med 3:106ra106, 2011) and “High-throughput DNA sequence analysis reveals stable engraftment of gut microbiota following transplantation of previously frozen fecal bacteria” (M. J. Hamilton, A. R. Weingarden, T. Unno, A. Khoruts, and M. J. Sadowsky, Gut Microbes 4:125–135, 2013), shaped how she approaches interpreting microbiome studies.

## Abstract (precis)

Anna M. Seekatz works in the field of the gut microbiome as it related to infectious diseases. In this “mSphere of Influence” article, she reflects on how two studies, “The impact of a consortium of fermented milk strains on the gut microbiome of gnotobiotic mice and monozygotic twins” (N. P. McNulty, T. Yatsunenko, A. Hsiao, et al., Sci Transl Med 3:106ra106, 2011) and “High-throughput DNA sequence analysis reveals stable engraftment of gut microbiota following transplantation of previously frozen fecal bacteria” (M.

## COMMENTARY

My favorite part about discovery is not the part that works, but the hidden story, the enigmatic result. Examining unexpected data is the engine of discovery. Throughout my training, two papers helped shape how I think about the gut microbiota, the indigenous microbes that inhabit the intestinal tract. Toward the end of my graduate studies, I read a paper by McNulty et al. entitled “The impact of a consortium of fermented milk strains on the gut microbiome of gnotobiotic mice and monozygotic twins” (1). This study beautifully laid out a translational method in a model system (germfree mice) to investigate how probiotic bacterial strains influence the gut ecosystem). Early on as a postdoctoral fellow, a second study, this one by Hamilton et al. (2), “High-throughput DNA sequence analysis reveals stable engraftment of gut microbiota following transplantation of previously frozen fecal bacteria,” implemented the more commonly used method of 16S rRNA sequencing to track the colonization of newly introduced bacteria from a fecal microbiota transplantation (FMT) in the gastrointestinal tracts of patients with *Clostridioides* (*Clostridium*) *difficile* infection (2). Both of these studies, while methodologically divergent, demonstrated measurable changes in the microbiome following the introduction of new microbes. However, to me, they also demonstrated that these changes occurred in an unexpected way, highlighting everything we do not know.

To investigate how probiotic strains impacted the human gut microbiota, McNulty et al. initially used both 16S rRNA sequencing and metagenomics to assess how bacteria in a fermented milk product (FMP) impacted the gut microbiota community in humans. Surprisingly, they did not observe a large impact of the strains on the community itself. This prompted the researchers to design a study in gnotobiotic mice: germfree mice inoculated with a defined community of sequenced, human-origin microbes were given the FMP, allowing for more precise tracking of potential changes in the microbiome. Metatranscriptomic sequencing of the gut microbiome in this simplified model demonstrated significant changes in the gene expression of carbohydrate active enzymes, despite a minimal impact on the microbiota community itself. The investigators then went back to the human cohort and were able to identify similar changes in this less defined and more complex environment.

The study by Hamilton et al. used solely 16S rRNA gene-based analysis to track transplantation of donor microbes from FMT used to treat recurrent Clostridioides difficile infection (CDI). FMT and reestablishment of a “healthy” microbiome has been demonstrated to be highly effective in treating CDI, and yet how the microbiome confers resistance is not clear. By tracking bacteria from the human donor in each of the three individuals over time, Hamilton et al. concluded that donor microbes could colonize recipients. However, the data also demonstrated that much of the community in the FMT recipient at later time points did not appear to originate from the donor directly.

Both of these studies influenced how I think about the introduction of new bacteria to an existing ecosystem within a host. Probiotic strains from FMP were observed to alter bacterial transcription of the community, whereas FMT induced an environment for establishing colonization by other beneficial microbes. The methodology used in McNulty et al. shaped the way I think about how we approach studying the gut microbiome. By using a simplified, defined microbial community of microbes with available sequenced genomes in a model system, the investigators were able to track specific changes in the microbiome and translate those findings to humans, a markedly more complex environment. While this was not the first germfree study conducted to investigate host-microbe interactions, it was the first study I had read that demonstrated a translational, “bench-to-bedside” approach to understanding a key observation that was not initially clear. Although perhaps not as sophisticated methodologically, the study by Hamilton et al. further challenged what I thought I knew about the gut microbiome. While the investigators concluded that transplantation of microbes as part of FMT did occur, what struck me was the individuality of the microbiome dynamics in each patient. What determined the trajectory toward a “recovered” microbiome, if not the donor microbes themselves? The combination of these two studies, as well as others since, has led me to think about what ecological and environmental factors determine how microbes colonize and interact.

As our knowledge about the importance of the human microbiome to disease has grown, so has our interest in developing methods to manipulate it for our benefit. Both knowledge and available methodology to study the microbiome has increased significantly since the publication of these studies. Multiple studies on the impact of FMT have now demonstrated that functional recovery of the microbiome to combat C. difficile is not necessarily accompanied by donor microbes; rather, the introduction of new bacteria as part of FMT may induce an environment conducive to colonization by less abundant beneficial microbes that already exist within a host or are introduced from the external environment (3, 4). A recent human study demonstrated immense variability in how the gut microbiome in different individuals responded to the consumption of probiotic strains (5). In a related study that investigated the impact of both probiotics and FMT on a gut microbiome exposed to antibiotic treatment, probiotic consumption was demonstrated to attenuate recovery of the preexisting community, presumably due to differences within the host (6). Both of these recent studies highlight how the extant microbiome shapes future colonization events. If we are to advance microbial manipulation for the benefit of human health going forward, it is critical to follow up on results that surprise us. In particular, there is a lack of characterization of the diverse, beneficial microbes observed to be important in health. How the host selects these microbes, or rather, how a microbe selects a host, is still intriguing to me. Furthermore, how these microbes behave in different environments, including in different individuals with their own unique microbiome and immune responses, has yet to be understood. Going forward as an independent investigator, these concepts will continue to drive my research, hopefully leading to new perspectives on how the human microbiome benefits us.

## References

[1] McNulty NP, Yatsunenko T, Hsiao A, Faith JJ, Muegge BD, Goodman AL, Henrissat B, Oozeer R, Cools-Portier S, Gobert G, Chervaux C, Knights D, Lozupone CA, Knight R, Duncan AE, Bain JR, Muehlbauer MJ, Newgard CB, Heath AC, Gordon JI. 2011. The impact of a consortium of fermented milk strains on the gut microbiome of gnotobiotic mice and monozygotic twins. Sci Transl Med 3:106ra106. doi:10.1126/scitranslmed.3002701. PMC3303609 22030749

[2] Hamilton MJ, Weingarden AR, Unno T, Khoruts A, Sadowsky MJ. 2013. High-throughput DNA sequence analysis reveals stable engraftment of gut microbiota following transplantation of previously frozen fecal bacteria. Gut Microbes 4:125–135. doi:10.4161/gmic.23571. 23333862 PMC3595072

[3] Smillie CS, Sauk J, Gevers D, Friedman J, Sung J, Youngster I, Hohmann EL, Staley C, Khoruts A, Sadowsky MJ, Allegretti JR, Smith MB, Xavier RJ, Alm EJ. 2018. Strain tracking reveals the determinants of bacterial engraftment in the human gut following fecal microbiota transplantation. Cell Host Microbe 23:229–240.e5. doi:10.1016/j.chom.2018.01.003. 29447696 PMC8318347

[4] Staley C, Kelly CR, Brandt LJ, Khoruts A, Sadowsky MJ. 2016. Complete microbiota engraftment is not essential for recovery from recurrent *Clostridium difficile* infection following fecal microbiota transplantation. mBio 7:e01965-16. doi:10.1128/mBio.01965-16. 27999162 PMC5181777

[5] Zmora N, Zilberman-Schapira G, Suez J, Mor U, Dori-Bachash M, Bashiardes S, Kotler E, Zur M, Regev-Lehavi D, Brik RB-Z, Federici S, Cohen Y, Linevsky R, Rothschild D, Moor AE, Ben-Moshe S, Harmelin A, Itzkovitz S, Maharshak N, Shibolet O, Shapiro H, Pevsner-Fischer M, Sharon I, Halpern Z, Segal E, Elinav E. 2018. Personalized gut mucosal colonization resistance to empiric probiotics is associated with unique host and microbiome features. Cell 174:1388–1405.e21. doi:10.1016/j.cell.2018.08.041. 30193112

[6] Suez J, Zmora N, Zilberman-Schapira G, Mor U, Dori-Bachas M, Bashiardes S, Zur M, Regev-Lehavi D, Ben-Zeev Brik R, Federici S, Horn M, Cohen Y, Moor AE, Zeevi D, Korem T, Kotler E, Harmelin A, Itzkovitz S, Maharshak N, Shibolet O, Pevsner-Fischer M, Shapiro H, Sharon I, Halpern Z, Segal E, Elinav E. 2018. Post-antibiotic gut mucosal microbiome reconstitution is impaired by probiotics and improved by autologous FMT. Cell 174:1406–1423.e16. doi:10.1016/j.cell.2018.08.047. 30193113

